# Book Review: Planning Later Life. Bioethics and Public Health in Ageing Societies

**DOI:** 10.3389/fsoc.2019.00022

**Published:** 2019-04-04

**Authors:** Marie-Kristin Döbler

**Affiliations:** Institute of Sociology, Friedrich-Alexander University of Erlangen-Nuernberg, Erlangen, Germany

**Keywords:** age, bioethic, public health, later life, book review, interdiciplinarity

Mark Schweda, Larissa Pfaller, Kai Brauer, Frank Adloff, Silke Schicktanz, (Oxon; New York, NY: Routledge), 2017, 264 pages, ISBN:978-1472481320.

Aiming at examining “the relevance of modern medicine and healthcare in shaping the future lives and situations of elderly people and aging societies,” the editors of “Planning Later Life” want to provide a comparative, multidisciplinary perspective on aging, later life and dealing with older people. The introduction by Schweda (medical ethicist), Pfaller (sociologist), and Schicktanz (bioethicist) provides an overview of the field, which is supposedly characterized by two dominant scenarios. First, there is the idea of a “self-determined and ‘successful’ aging,” which can and should be “prudently modeled and actively shaped.” This corresponds with the individualisation of responsibilities and subtle, or even quite explicit, forms of pressures: people are requested to invest into provisions, and despite aging, to continue to be healthy. The second scenario centers around chronic diseases, frailty, dementia, and envisioned threats posed thereby toward collectives. United by their normative criticisms of currently dominant concepts and their imaginaries about aging and old age, some contributions problematize the consequences of the (continued) application of narratives and subjectivation associated with either of the mentioned scenarios. Some chapters discuss the juxtaposition of assumed degrees of self-responsibility and consequences of different forms of individualisation in the context of being or getting old with political and societal challenges for dealing with associated demographic changes and an assumed decline in intergenerational solidarity. Finally, most chapters try to promote alternative perspectives and concepts on age(ing). Overall the anthology amounts to a polyphone account against the fatalistic view, which is connected with practices of victim-blaming and discriminatory agist discourses, and against the first scenario inspired by neoliberal, market-based thinking.

Following Mark Schweda, a life-course perspective has proven to be fruitful in understanding bio-ethical and health issues. Accordingly, the temporality and vulnerability of human existence can be recognised without or instead of artificially differentiating ethics of young and old people. By tracing ideas about aging back to the ancient ethics of the good life, Thomas Rentsch deepens the philosophical considerations indicated in Schweda's elaborations. Subsequently, François Höpflinger continues to deal with age distinctions. From a sociological stance he problematises the thinking in terms of a “third” and a “fourth age,” related to different images, treatments and policies which parallel the scenarios previously introduced. Paul Higgs and Chris Gilleard focus on the negative attributions to “fourth age” and the social implications of this “imaginary,” coined by associated individuals' fears which are societally spread.

Chapters of the second part operate in front of this conceptual background, but concentrate more on particular phenomenon and problems of old age. Beginning with a gerontologist's view, Andreas Kruse argues that ethical and social science approaches should take account of both, vulnerability and development potentials even in their considerations of very old age, dementia and care. Perla Werner and Silke Schicktanz deal with ethical questions on dementia, which should be addressed more in face of growing numbers of people suffering from this form of mental decay and the increasing number of scientific measurements of their competence. Subsequently, Hsiu-I Yang suggests that in case of very old people it might be necessary and human to exchange the idea of sustaining the life for any price with considerations of a peaceful death. Søren Holm considers the ethical implications of biomedical life extension technologies, while Nancy Jeckers expands on the problems connected to the wide-spread interpretation of rising health care costs as a burden for younger generations.

Kai Brauer starts the anthology's third part with a discussion of decisions people make in anticipation of unpredictable future events, materialised e.g. in living wills. Ralf J. Jox deepens these considerations with his views on advance healthcare planning. The next chapter, by S. Jay Olshansky, deals with medical and public health strategies of prevention and longevity. Olshansky portraits technological and medical practices that slow down aging in people as plausible targets for science and public health in order to combat disease and morbidity. Larissa Pfaller and Frank Adloff take a more critical stance in tackling practices of anti-aging and prevention in Germany. Likewise, Silke Schicktanz concentrates on different forms of power, socio-cultural influences on the individual and the impacts of normatively coined prescriptions of successful aging. Some parallels to Schicktanz' elaborations on the societal dimensions of diagnosing and caring for people with dementia can be found in Ruud ter Meulen's discussion of public health perspectives on care for the elderly. Problematizing the advance of neoliberal considerations in the context of aging, he fears the decline of social solidarity and introduces a case-study on the network-like cooperation of family and professional care givers practiced in the Netherlands. The book ends with Stephen Katz and Peter J. Whitehouse's portray of The Intergenerational Schools (TIS): learning communities and ethical models of intergenerativity founded in America.

In sum, the edited volume “Planning Later Life” draws a multifaceted picture of aging, later life and dealing with older people. Often dedicated to a certain disciplines and focused on case-studies from Europe and the US, the 16 chapters shed light on central issues, controversially debated in public and within the scientific community. Complemented by occasional references to Asia, the volumes targeted internationality is partly met, even if the majority of the contributions maintain a Western, Eurocentric view, limiting the cultural diversity acknowledged. Nevertheless, in conjunction, the different contributions offer a broad, diverse overview on envisioning, evaluating, and controlling “later life.” Beginning with the introduction, the anthology (1) pays attention to relevant ethical, moral, and political dimensions in the context of aging; (2) highlights, in a diverse way, the various connections with social theories and macro structural developments or changes; (3) and critically reviews the effects of advances in medicine and healthcare and their ambivalent, or even contradictory, interpretations, which often sediment in medially spread and politically instrumentalized scenarios.

## Author Contributions

The author confirms being the sole contributor of this work and has approved it for publication.

### Conflict of Interest Statement

The author declares that the research was conducted in the absence of any commercial or financial relationships that could be construed as a potential conflict of interest.

